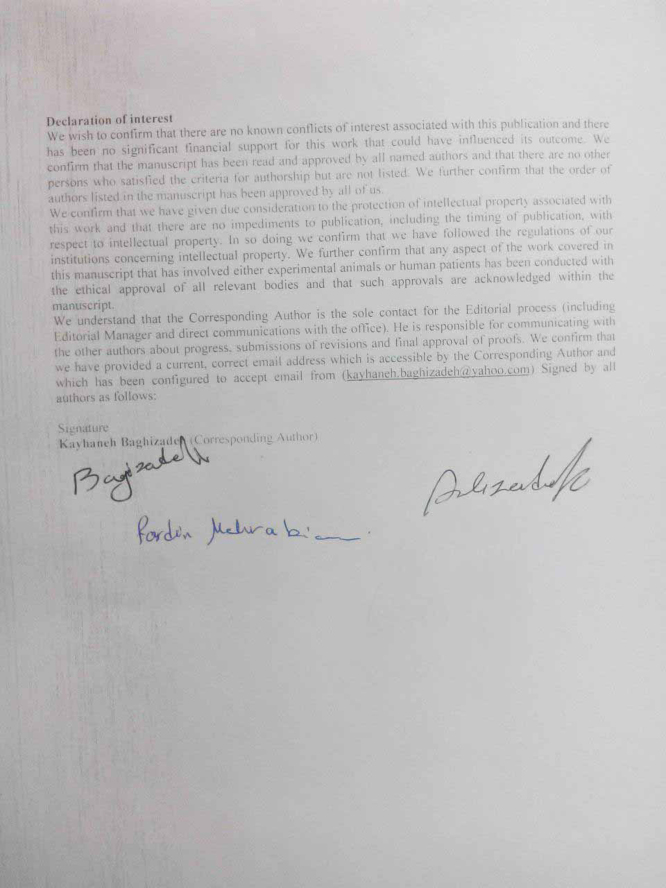# The relationship between empowerment, occupational burnout, and job stress among nurses in Rasht Medical Education Centers: A dataset

**DOI:** 10.1016/j.dib.2018.08.176

**Published:** 2018-09-01

**Authors:** Fardin Mehrabian, Kayhaneh Baghizadeh, Iman Alizadeh

**Affiliations:** a Research Center of Health and Environment, School of Health, Guilan University of Medical Sciences, Rasht, Iran; b School of Health, Guilan University of Medical Sciences, Rasht, Iran; c Islamic Azad University, Lahijan Branch, Lahijan, Iran; d Department of English Language Teaching, School of Nursing, Midwifery, and Paramedical Sciences, Guilan University of Medical Sciences, Rasht, Iran

**Keywords:** Empowerment, Occupational burnout, Job stress, Nurses

## Abstract

The data in this article show the relationship between empowerment, occupational burnout and job stress among nurses in medical education centers in the city of Rasht, Iran. This descriptive correlational study was carried out from January 2016 to June 2017. The study sample included 316 nurses working at the teaching hospitals in Rasht; they were selected using stratified random sampling. The data were collected by a standard questionnaire which was rated based on the five-point Likert Scale. Data analysis was performed by SPSS-22 and SmartPLS software. Data analyzing showed that the mean scores of occupational burnout and job stress were lower than the average level. There was a significant relationship between empowerment and job stress and between job stress and occupational burnout. However, there was no significant association between empowerment and occupational burnout. It was discovered that job stress played a mediating role on the relationship between empowerment and occupational burnout.

**Specifications Table**

**Table:** 

**Subject area**	Nurses in Rasht Medical Education Centers
**More specific subject area**	Describe narrower subject area
**Type of data**	Table
**How data was acquired**	Data were collected by a four-part standard questionnaire. The first section of the questionnaire was on demographic information including gender, age, marital status, education and work experience; the second section (items 1–18) focused on structural empowerment; the third section (items 19–30) analyzed job stress and the fourth section (items 31–52) explored occupational burnout.
**Data format**	Raw, analyzed
**Experimental factors**	Scoring was performed based on the five-point Likert Scale. The validity of the scale was examined and confirmed using the views of experts in the field; its reliability was also confirmed by Cronbach׳s alpha.
**Experimental features**	The normal distribution of the data was analyzed and approved by Kolmogorov-Smirnov test. SPSS-22 software was used to analyze the descriptive statistics, and because of the low sample size, PLS2 software or least squares approach was used to analyze the fit of the model and to test the research hypotheses.
**Data source location**	Rasht, Guilan Province, Iran.
**Data accessibility**	The data are available with this data article and its tables.

**Value of the data**

• Nurses, who play a key role in health organizations, experience high levels of job stress and burnout, which might be due to their low or limited capabilities. Moreover, an increase in the level of occupational burnout and stress can adversely affect productivity and efficiency.
• Occupational burnout and job stress are among the main problems with which many health organizations are burdened.
• The data shown here can be used for the health organizations and medical education centers managers.

## 1 Data

Various factors affect the improvement of manpower productivity [1], [2], [3]. Any organization which provides opportunities for its staff members to gain higher levels of capability is more able to face challenges, guarantees its long-term sustenance [4], and aids its staff having a better work quality and performance [5]. An increase in job stress decreases the motivation for work and, as a consequence, in productivity [6], [7].

### 1.1 Demographic characteristics

The results in this part showed that 295 nurses (93.4%) were female and 21 (6.6%) were male, of whom 130 (41.1%) were single and 186 (58.9%) were married; 75 (23.7%) had postgraduate education, 222 (70.3%) had a bachelor׳s degree and 19 (6%) had an associate degree. In addition, 91 nurses (28.8%) were aged <30 years, 121 (38.3%) were aged 31–40, 81 (25.6%) were aged 41–50 years and 23 (7.3%) were aged >50 years. Furthermore, 66 (20.9%) nurses had 1–5 years, 94 (29.7%) had 6–10 years, 85 (26.9%) had 11–15 years, 58 (18.4%) had 16–20 years and 13 (4.1%) had >20 years of work experience.

The results of empowerment, job stress, and occupational burnout sections of the questionnaire were as follows:

Empowerment: the minimum score was 1.56, the maximum score was 5, the mean score was 3.65, the standard deviation was 0.65709 and variance was 0.432.

Job stress: the minimum score was 1.00, the maximum score was 4.50, the mean score was 2.47, the standard deviation was 0.63282 and variance was 0.400.

Job burnout: the minimum score was 1.20, the maximum score was 4.41, the mean score was 2.51, the standard deviation was 0.52501 and variance was 0.276.

In general, the findings indicated that the mean score of empowerment was higher than the expected mean [4] and the mean scores of occupational burnout and job stress were lower than the expected mean.

### 1.2 The findings of the study indicated

1. The relationship between empowerment and job stress;

The *t*-test value on the relationship between structural empowerment and job stress was out of the range (−1.96 and 1.96); hence, the hypothesis was accepted. The association level between structural empowerment and job stress was equal to −0.345 (Table 1).

2. The relationship between empowerment and occupational burnout

**Table 1:** Results of the analysis of the first research hypothesis.

**Research hypothesis**				***T*-value**	**Path coefficient**	**Result**
H_1_	Structural empowerment	→	Job stress	10.768	−0.345	Confirmed

The *t*-test value on the relationship between structural empowerment and occupational burnout was within the range (−1.96 and 1.96); thus, the hypothesis was rejected (Table 2).

3. The relationship between job stress and occupational burnout

**Table 2:** Results of analysis of the second research hypothesis.

**Research hypothesis**				***T*-value**	**Path coefficient**	**Result**
H_2_	Structural empowerment	→	Job burnout	0.079	−0.007	Rejected

Considering the structural model of the study for the significant coefficient, the *t*-test value on the relationship between empowerment and job stress was out of the range (−1.96%1.96); hence, the hypothesis was accepted. The relationship level between job stress and occupational burnout was equal to −0.886 (Table 3).

4. The mediating role of job stress on the relationship between empowerment and occupational burnout

**Table 3:** Results of the analysis of the third research hypothesis.

**Research hypothesis**				***T*-value**	**Path coefficient**	**Result**
H_3_	Job stress	→	Job burnout	10.768	−0.866	Confirmed

To analyze the mediating role of job stress on the relationship between structural empowerment and occupational burnout, Sobel test was used.

$$z-\mathrm{Value}=\frac{a*b}{\sqrt{{(b}^{2}*s_{a}^{2})+{(a}^{2}*s_{b}^{2})+{(s}_{a}^{2}*s_{b}^{2})}}$$

In this equation:

*a*: path coefficient between independent and mediator variables
*b*: path coefficient between the mediator and dependent variables
*S_a_*: standard error of independent and mediator variables
*S_b_*: standard error of mediator and dependent variables
  Z=3.343

The findings showed a *z* value of >1.96 showing that job stress had a mediating role on the relationship between structural empowerment and occupational burnout. Considering the coefficients between structural empowerment and job stress and between job stress and occupational burnout, it can be said that the relationship between structural empowerment and occupational burnout, with the mediating role of job stress, was equal to 0.298.

## 2 Experimental design, materials and methods

This descriptive correlational study was carried out from January 2016 to June 2017. The study population consisted of 1586 nurses working in Alzahra, Poursina, Razi, Dr. Heshmat, 17Shahrivar, Amiralmomenin, Shafa and Velayat hospitals, who were selected using Cochran׳s sample size formula:

$$n=\frac{Z_{\frac{a}{2}}^{2}\cdot S_{X}^{2}\cdot N}{e^{2}\cdot N-e^{2}+\left(Z_{\frac{a}{2}}^{2}\cdot S_{X}^{2}\right)}$$

A sample of 316 nurses was obtained from the calculations and questionnaires were distributed in each hospital using stratified random sampling, as presented in Table 4 below.

**Table 4:** Distribution of questionnaires in Rasht hospitals.

**Hospital**	**Number of nurses**	**Required sample**	**Distributed number**	**Collected number**
**Alzahra**	123	24	32	25
**Poursina**	355	69	90	71
**Razi**	317	62	81	62
**Heshmat**	228	44	57	42
**17 Shahrivar**	183	36	47	37
**Amiralmomenin**	84	17	22	18
**Shafa**	180	35	46	36
**Velayat**	116	23	30	25
**Total**	1586	310	405	316

Data were collected by a four-part standard questionnaire. The first section of the questionnaire was on demographic information including gender, age, marital status, education and work experience; the second section (items 1–18) focused on structural empowerment [8]; the third section (items 19–30) analyzed job stress [9], and the fourth section (items 31–52) explored occupational burnout [10]. Scoring was performed based on the five-point Likert Scale. The validity of the scale was examined and confirmed using the views of experts in the field; its reliability was also confirmed by Cronbach׳s alpha (Table 5).

**Table 5:** Cronbach׳s alpha coefficient for reliability of the questionnaire items.

**Dimensions**	**α**	**Result**
**Structural empowerment**	0.897	Confirmed
**Job stress**	0.882	Confirmed
**Job burnout**	0.908	Confirmed

The normal distribution of the data was analyzed and approved by Kolmogorov-Smirnov test. SPSS-22 software was used to analyze the descriptive statistics, and because of the low sample size, PLS2 software or least squares approach was used to analyze the fit of the model and to test the research hypotheses.

## Figures and Tables

**Fig. S1:**